# Starvation Increases Insulin Sensitivity and Reduces Juvenile Hormone Synthesis in Mosquitoes

**DOI:** 10.1371/journal.pone.0086183

**Published:** 2014-01-29

**Authors:** Meritxell Perez-Hedo, Crisalejandra Rivera-Perez, Fernando G. Noriega

**Affiliations:** Department of Biological Sciences, Florida International University, Miami, Florida, United States of America; New Mexico State University, United States of America

## Abstract

**Background:**

The interactions between the insulin signaling pathway (ISP) and juvenile hormone (JH) controlling reproductive trade-offs are well documented in insects. JH and insulin regulate reproductive output in mosquitoes; both hormones are involved in a complex regulatory network, in which they influence each other and in which the mosquito's nutritional status is a crucial determinant of the network's output. Previous studies reported that the insulin-TOR (target of rapamacyn) signaling pathway is involved in the nutritional regulation of JH synthesis in female mosquitoes. The present studies further investigate the regulatory circuitry that controls both JH synthesis and reproductive output in response to nutrient availability.

**Methods:**

We used a combination of diet restriction, RNA interference (RNAi) and insulin treatments to modify insulin signaling and study the cross-talk between insulin and JH in response to starvation. JH synthesis was analyzed using a newly developed assay utilizing fluorescent tags.

**Conclusions:**

Our results reveal that starvation decreased JH synthesis via a decrease in insulin signaling in the *corpora allata* (CA). Paradoxically, starvation-induced up regulation of insulin receptor transcripts and therefore “primed” the gland to respond rapidly to increases in insulin levels. During this response to starvation the synthetic potential of the CA remained unaffected, and the gland rapidly and efficiently responded to insulin stimulation by increasing JH synthesis to rates similar to those of CA from non-starved females.

## Introduction

The correct allocation of nutrients between conflicting needs such as reproduction, growth, maturation or flight is a vital component of an insect's life-history strategy [1]. Juvenile hormone (JH) is a remarkably versatile molecule with major effects on various aspects of development and life history in insects, including metamorphosis, behavior, reproduction, diapause, stress resistance and aging [2]. The insulin signaling pathways (ISP) is an evolutionary conserved systemic nutritional sensor that plays a central role in the transduction of nutritional signals that regulate cell growth and metabolism [3], [4]. JH and insulin are involved in a complex regulatory network, in which they influence each other and in which the insect's nutritional status is a crucial determinant of the network's output [5].

The reproductive cycle of female mosquitoes proceeds through a previtellogenic stage (PVGS), an ovarian resting stage (ORS) and a vitellogenic stage (VGS) [6]. In *Aedes aegypti* JH is a key mediator of trade-offs between survival and reproduction during the PVGS and ORS [7], [8]. Regardless of the nutritional status of a female mosquito, topical application of JH will override other signaling pathways and stimulate oogenesis even in isolated abdomens [9], [7]. In addition, increased insulin production and signaling, in response to a positive nutritional status, tends to stimulate egg production in mosquitoes [10].

How these two hormonal signaling pathways (ISP and JH) interact to regulate all the physiological functions necessary for egg production is not totally clear. JH titers in female adult mosquitoes are mainly determined by the rate at which the *corpora allata* (CA) synthesize JH [11]. Changes in JH synthesis in female adult *A. aegypti* mosquito are very dynamic and nutrition-dependent [12]. Previous studies reported that the insulin/target of rapamaycin (TOR) pathway is involved in the nutritional regulation of JH synthesis in female mosquitoes [13]. Application of bovine insulin on the mosquito CA-CC incubated *in vitro* caused a strong and fast stimulation on JH synthesis; while systemic depletion of TOR by RNAi and rapamycin administration had inhibitory effects on JH synthesis [13]. In addition, reducing insulin signaling with PI3K inhibitors rapidly reduced JH biosynthetic enzyme transcripts; validating that a transcriptional regulation of the genes encoding JH biosynthetic enzymes is at least partially responsible for the dynamic changes of JH biosynthesis in mosquitoes [13].

Here diet restriction, *in vivo* depletion of INSr and FOXO using RNA interference (RNAi) and insulin treatments were used to modify insulin signaling and study the cross-talk between insulin and JH in response to starvation. Our results reveal that starvation decreased JH synthesis via a decrease in insulin signaling in the CA. Starvation-induced up regulation of the insulin receptor increased CA insulin sensitivity and might “prime” the gland to respond rapidly to increases in insulin levels after feeding resumption. During this response to starvation the synthetic potential of the CA remained unaffected, and the gland rapidly and efficiently responded to insulin stimulation by increasing JH synthesis to rates similar to those of CA from non-starved females.

## Materials and Methods

### Insects

*Aedes aegypti* of the Rockefeller strain were reared at 28°C and 80% relative humidity under a photoperiod of 16 h light: 8 h dark. Female were offered a cotton pad soaked in 20% sucrose solution for the first 24 h after adult emergence. Afterwards, one day-old females were separated into two experimental groups: starved females were offered water, and sugar-fed females were maintained on a 20% sucrose solution. All experiments were performed with 4-day old virgin females.

To test the effect of starvation we used four days old females because our RNAi protocol requires waiting a 4-day period after injection to be effective [13]. Starving newly emerged females for 4 days resulted in an elevated mortality. Feeding females on a high sugar meal (20% sucrose) for 24 h before starving them for 3 days improved survival to almost 100%. In the rest of the manuscript we will call “sugar-fed” to those females fed 20% sugar for 4 days and “starved” or “water-fed” to those fed water from day 2 to 4.

### Lipid assays

Lipids were quantified in mosquitoes using a triglyceride quantification kit (K622-100, Biovision, Mountain View, CA) as previously described [8].

### Assessment of *in vitro* JH production

*Corpora allata-corpora cardiaca* complexes (CA-CC) attached to the head capsule were isolated from adult females as previously described [11]. JH synthesis was evaluated on CA-CC incubated at 32°C in M-199 medium (Lavallette, NJ, USA) containing 2% Ficoll, 25 mM HEPES (pH 6.5) and methionine (50 mM). Bovine insulin was purchased from Akron Biotech (Boca Raton, FL), dissolved in HCl and used at 17 µM, a concentration previously established to be optimal [13].

### Quantification of JH synthesis

The amount of JH synthesized by CA-CC complexes *in vitro* was quantified using high performance liquid chromatography coupled to a fluorescent detector (HPLC-FD) [14]. The assay is based on the derivatization of JH III with a fluorescent tag with subsequent analysis by reverse phase HPLC-FD.

### RNA extraction, reverse transcription and real-time PCR

Total RNA was isolated using RNA-binding glass powder as previously described [15]. Contaminating genomic DNA was removed using Turbo DNA-free DNase (Ambion, Austin, TX, USA). Reverse transcription was carried out using an oligodT priming method and SuperScrit® III First-Strand Synthesis System Kit (Invitrogen, Carlsbad, CA, USA). qPCR was performed with anApplied Biosystems7300 Real Time PCR System using TaqMan® Gene Expression Assays together with TaqMan® Universal PCR Master Mix (Applied Biosystems, Foster City, CA, USA). PCR reactions were run in triplicate using 1 µl of cDNA per reaction in a 20 µl volume according to manufacturer recommendations for Custom TaqMan® Gene Expression Assays. To obtain absolute transcript values, standard curves were made from serial dilutions of plasmids containing the mosquito genes (300,000; 30,000; 3,000; 300; 30 copies of a plasmid per reaction). Real-time data were collected by the 7300 System SDS Software and analyzed in Microsoft Excel. Transcript levels were normalized with rpL32 transcript levels in the same sample. Each qPCR data point is the average of 4 to 6 independent experiments analyzing either three groups of ten CA or three thoraxes. The primer probes sequences and accession numbers for the genes studied, insulin receptor (INSr), Forkhead-box-binding protein (FOXO), target of rapamycin (TOR), translation initiation inhibitor eIF4E-binding protein (4E-BP) and juvenile hormone acid methyltransferase (JHAMT); as well as the housekeeping gene 60S ribosomal protein L32 are included in Table S1.

### RNA interference

Synthesis and microinjections of double-stranded RNA (dsRNA) were performed as described by Perez-Hedo et al. [13]. INSr, FOXO and YFP (yellow fluorescent protein) target sequences for dsRNA synthesis were amplified by PCR using the INSri, FOXOi and YFPi primers (Table S1). The resulting amplicons were diluted 50-fold, and 1 µl of these diluted amplicons were used as template in PCR reactions with primers containing T7 promoter sequences (INSri _T7, FOXOi _T7 and YFPi_T7) (Table S1). The products from these PCR reactions were purified using a QIAquick PCR purification kit (QIAquick sciences, Germantown, Maryland), and 1–2 µg of the purified DNA templates were used to synthesize dsRNAs with a Megascript RNAi kit (Ambion, Austin, TX). dsRNAs were precipitated using ammonium acetate/ethanol, and resuspended in ddH2O to a final concentration of 3–4 µg/ml. In each knockdown experiment, newly emerged female mosquitoes were cold anesthetized and injected intra-thoracically with 1.6 µg of dsRNA using a Drummond Nanoject II microinjector and a micromanipulator. The effect of dsRNA was evaluated 4 days after injection. YFP was used as a negative control.

### Statistical analysis

Statistical analyses were performed using the GraphPad Prism Software (San Diego, CA, USA). The results are expressed as means ± S.E.M. Significant differences (P<0.05) were determined with a one tailed students t-test performed in a pair wise manner or one-way ANOVA followed by a comparison of means (Tukey's test).

## Results

### Starvation decreases mosquito lipid reserves and reduces JH synthesis

**Figure 1:**
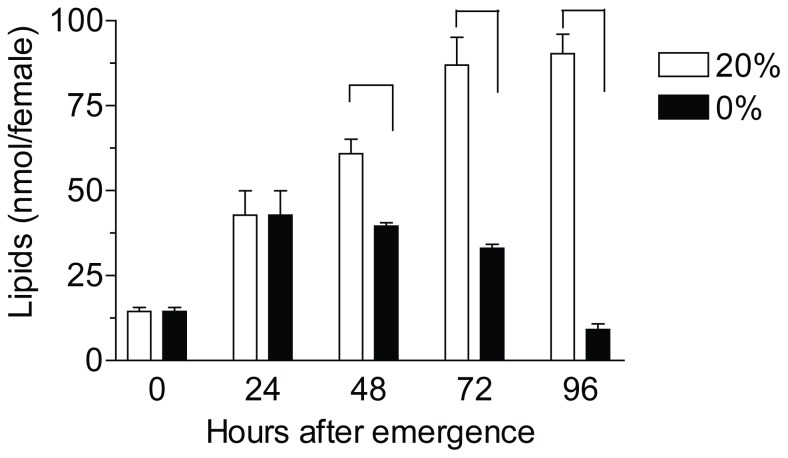
Starvation decreases mosquito lipid reserves. The total amount of lipids in the female body significantly increased when mosquitoes were fed 20% sucrose as compared to those fed water alone (0%). Bars represent the means ±SEM of 3 groups of 5 females per treatment. Asterisks denote significant differences between the two treatments for each time point (unpaired t-test, **P<0.01, *P<0.05).

To determine the effect of starvation on lipid reserves, the total lipid contents of mosquitoes were quantified in either sugar-fed or starved females. Lipids reserves increased more than 2-fold by feeding 20% sugar for the first 24 h. Lipids continued to increase in sugar-fed females, while starved females gradually lost lipids during the starvation period. There was a 10 fold decrease in total lipid contents of 4 day-old starved females when compared with mosquitoes fed 20% sucrose (Fig. 1).

**Figure 2:**
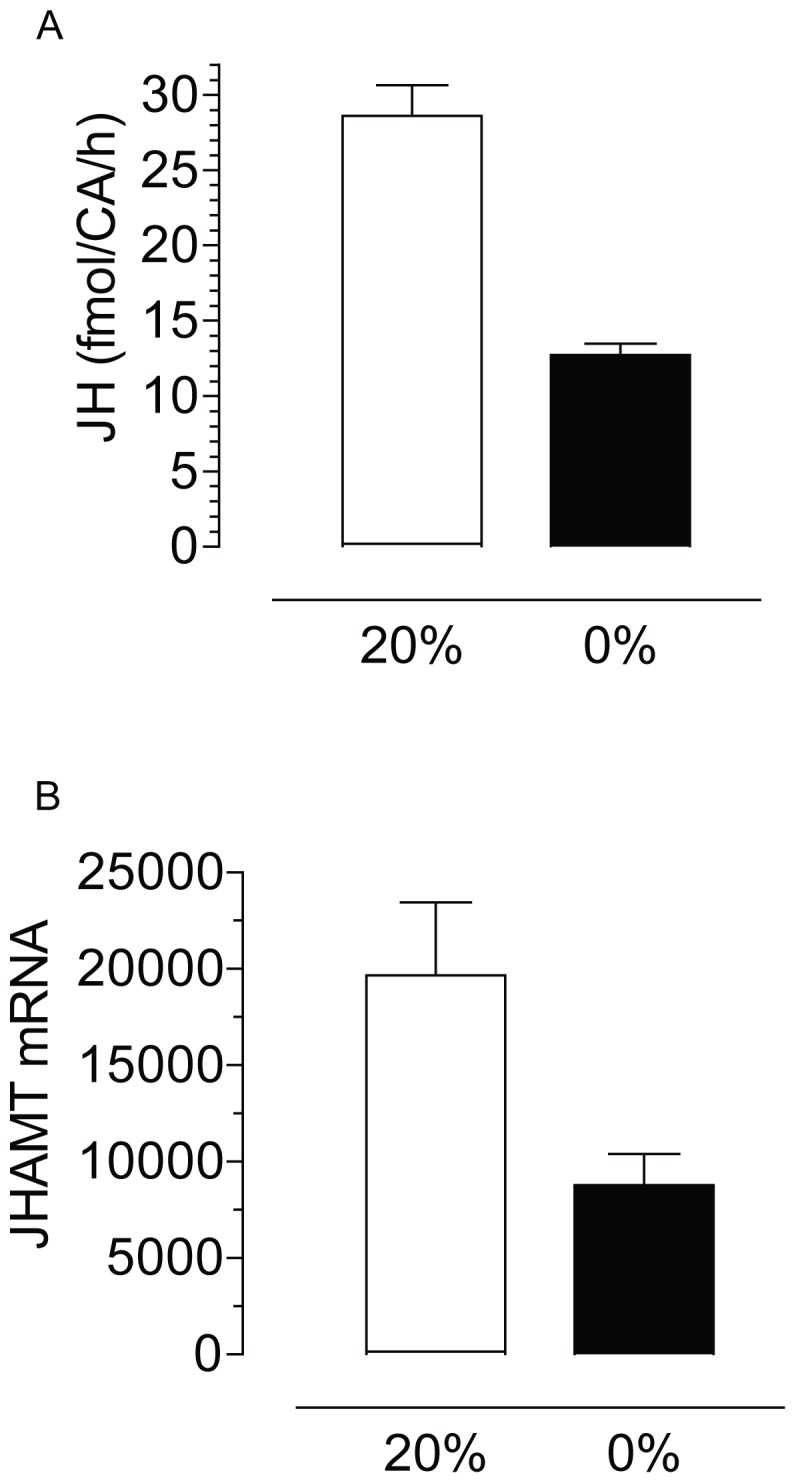
Starvation decreases JH synthesis and JHAMT transcript levels. CA-CC complexes were dissected from 4 days old females after 3 days feeding water (0%) or 20% sucrose (20%). A) JH synthesis: groups of 3 glands were incubated for 4 h; JH synthesis was evaluated by HPLC-FD and it is expressed as fmol per CA per hour. Bars represent the means ± S.E.M. of the analysis of 7 independent experiments of 5 groups of 3 glands per treatment. Different letters above the columns indicate significant differences among treatments (unpaired t-test, ***P<0.001). B) JHAMT transcript levels: CA-CC complexes were dissected from 4 days old females fed on water or 20% sucrose. Transcript levels were measured by qPCR and are expressed as copy number of mRNA JHAMT/10,000 copies of rpL32 mRNA. Bars represent the means ± S.E.M. of 10 replicates of groups of 10 CA each (unpaired t-test, *P<0.05).

Starvation resulted in a significant decrease of JH synthesis (Fig. 2A). The CA of 4-day old females starved for 72 h exhibited less than 45% of the synthetic activity of those glands from females fed 20% sugar. To further understand the role of starvation on JH synthesis we studied its effect on the expression of juvenile hormone acid methyltransferase (JHAMT), a key JH biosynthetic enzyme. We observed a statistically significant reduction in JHAMT transcript levels in the CA of starved adult female mosquitoes (Fig. 2B).

### Starvation modulates the expression of insulin/TOR pathway genes

**Figure 3:**
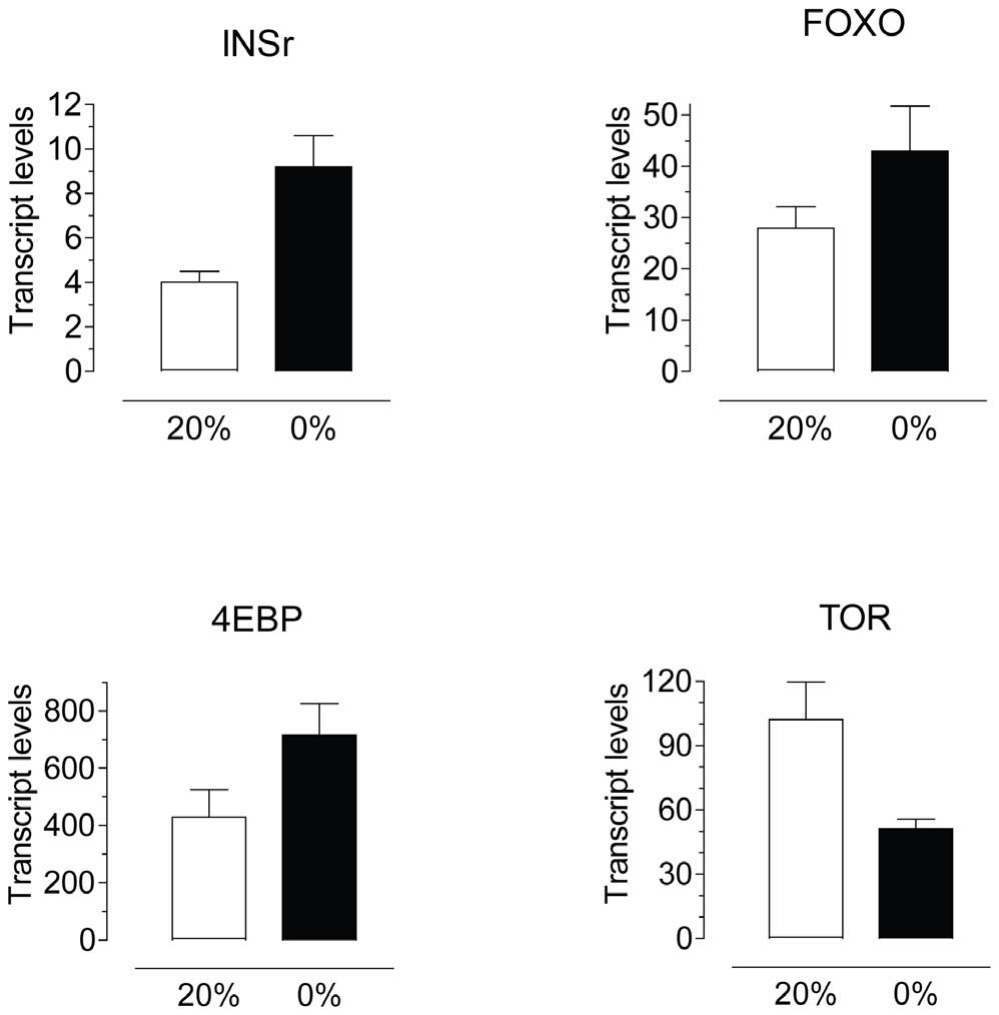
Starvation modulates the expression of insulin/TOR pathway genes. CA-CC complexes were dissected from 4 days old females after 3 days feeding water (0%) or 20% sucrose (20%). Insulin receptor (INSr), forkhead-box 0 (FOXO), 4E-binding protein (4EBP) and target of rapamycin (TOR) transcript levels were measured by qPCR and are expressed as copy number of mRNA gene/10,000 copies of rpL32 mRNA. Bars represent the means ± S.E.M. of 6 replicates of 10 CA each (unpaired t-test, ***P<0.001, **P<0.01, *P<0.05).

To dissect the transcriptional regulatory circuitry of the insulin signaling cascade in the CA in response to starvation, transcript levels for 4 key genes were analyzed in the CA of 4 days old adult females fed sugar or water (Fig. 3).Transcript levels for the insulin receptor (INSr), the Forkhead-box-binding protein (FOXO) and the translation initiation inhibitor eIF4E-binding protein (4EBP) were significantly increased in the CA of starved females. Transcripts for the target of rapamycin (TOR) protein were significantly decreased in the CA of starved females.

### *In vivo* depletion of insulin receptor or FOXO modifies JH synthesis

**Figure 4:**
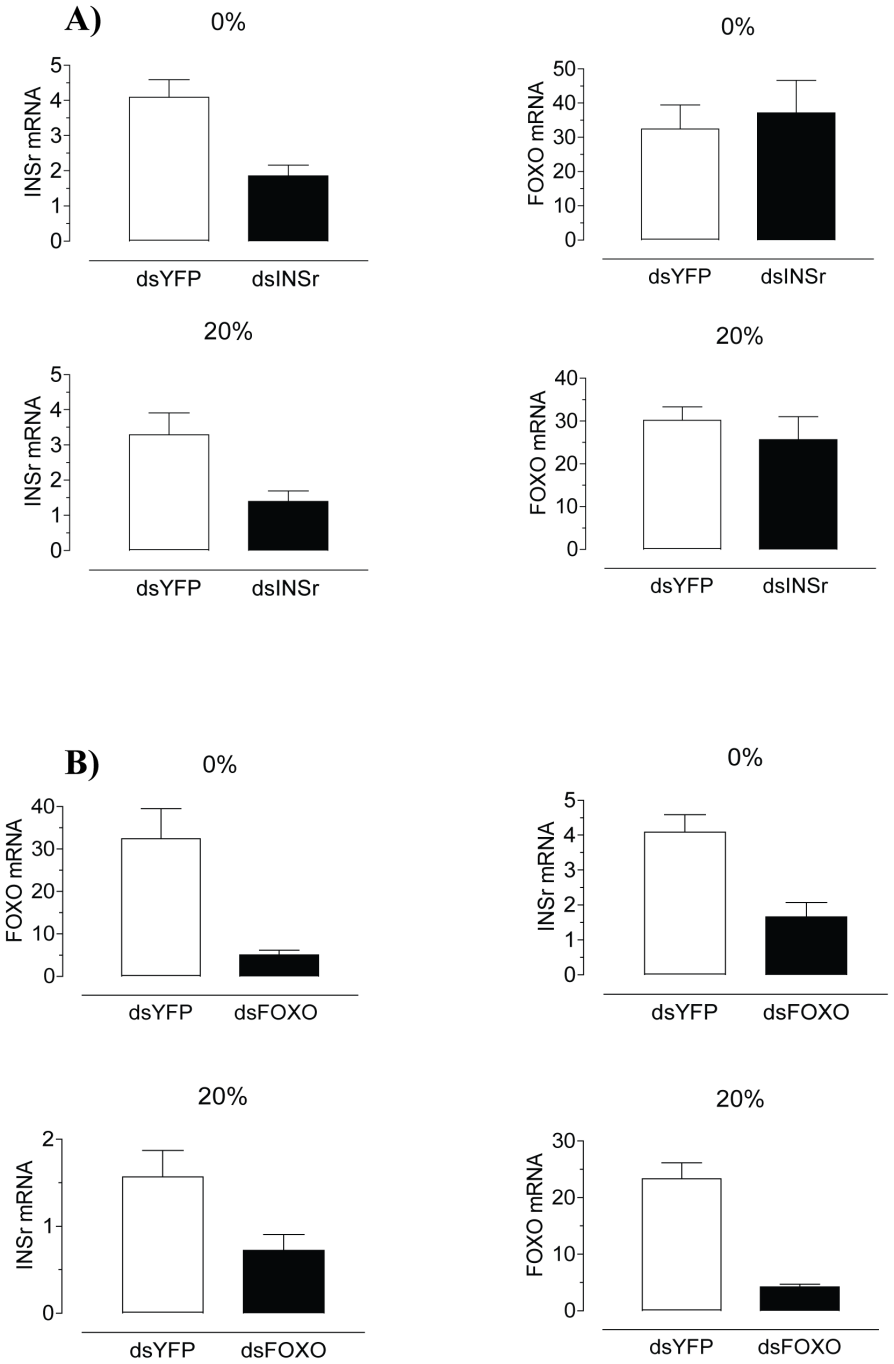
Effects of dsRNA treatment on INSr and FOXO transcript levels. Newly emerged females were injected with dsYFP, dsINSr or dsFOXO. The effect of the RNAi treatment was evaluated 4 days later. A) Effect of dsINSr treatment. B) Effect of dsFOXO treatment. The expression of INSr and FOXO mRNA in the thorax (without legs and wings) were evaluated by qPCR, and it is expressed as copy number of mRNA INSr or FOXO/10,000 copies of rpL32 mRNA. Bars represent the means ± S.E.M. of 3 independent biological replicates of 3 groups of 3thoraces. Asterisks denote significant difference (unpaired t-test, ***P<0.001,**P<0.01, *P<0.05).

To further understand the role of insulin pathway components on JH synthesis we analyzed the effect of *in vivo* depletion of INSr and FOXO. Injection of dsINSr resulted in a significant reduction of INSr mRNA when compared with dsYFP treated in both starved (55%) and sugar-fed adult females (58%), without reducing FOXO's transcript levels (Fig. 4A). In contrast, injection of dsFOXO resulted in significant reductions of both FOXO (82–85%) and INSr mRNAs (55–60%) in starved and sugar-fed adult females when compared with dsYFP treated controls (Fig. 4B).

**Figure 5:**
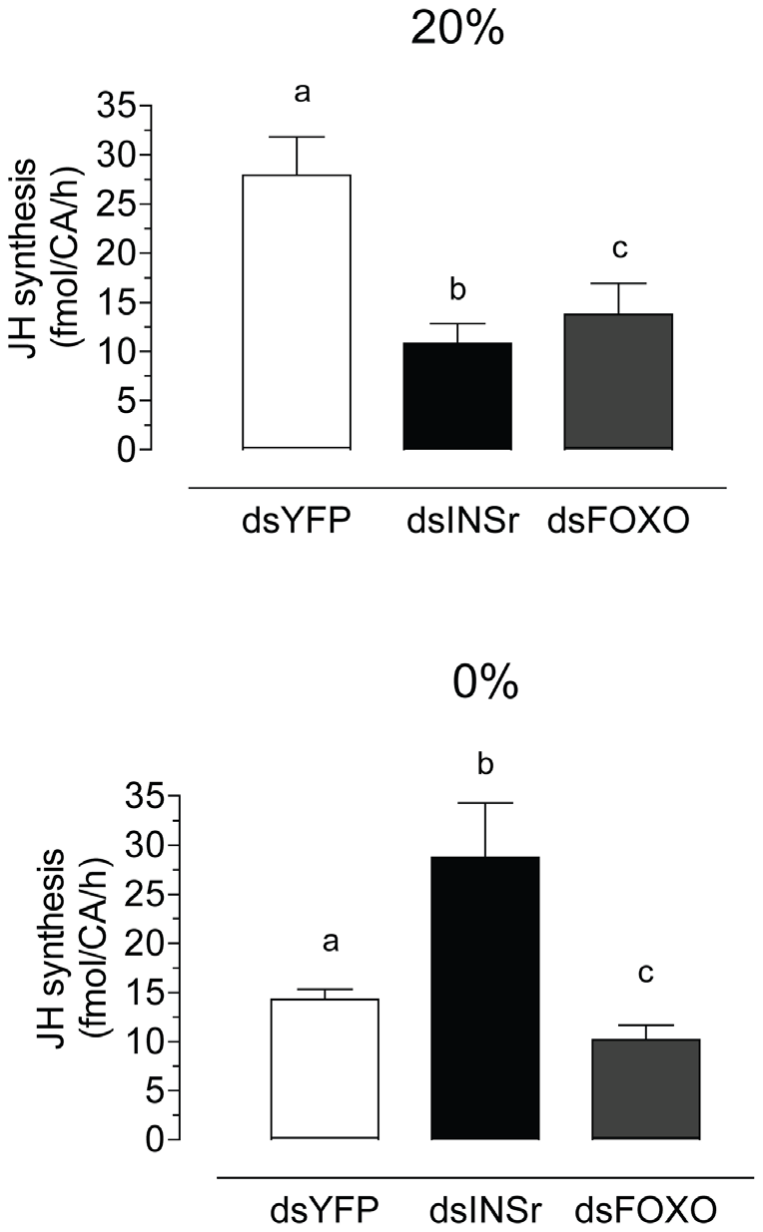
Effect of RNAi silencing of INSr and FOXO on JH synthesis. Newly emerged females were injected with dsYFP, dsINSr or dsFOXO. CA-CC complexes were dissected from 4 days old females after 3 days feeding water (0%) or 20% sucrose (20%). Groups of 3 glands were incubated for 4 h and JH synthesis was evaluated by HPLC-FD, and it is expressed as fmol per CA per hour. Bars represent the means ± S.E.M. of the analysis of 6–10 independent experiments of groups of 3 glands per treatment. Different letters above the columns indicate significant differences among treatments (one way ANOVA p<0.05, with Tukey test of multiple comparisons).

Systemic depletion of FOXO mRNA caused a significant reduction of JH synthesis in sugar-fed and starved females (Fig. 5).On the contrary, depletion of INSr mRNA had opposite effects depending on the nutritional status of mosquitoes; it decreased JH synthesis in sugar-fed females and increased JH synthesis in starved females (Fig. 5).

### Starvation increases CA insulin sensitivity

**Figure 6:**
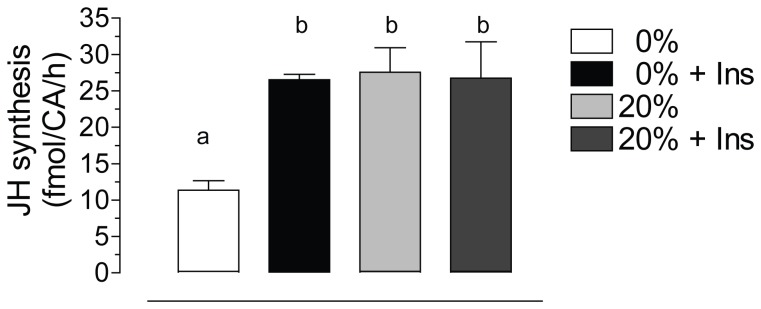
Starvation increases CA insulin sensitivity. CA-CC complexes were dissected from 4 days old females after 3 days feeding water (0%) or 20% sucrose (20%). Glands were incubated for 4 h in the presence of 17 mM of bovine insulin (+ Ins) or in medium alone. JH synthesis was evaluated by HPLC-FD, and it is expressed as fmol per CA per hour. Bars represent the means ± S.E.M. of the analysis of 3 independent experiments of 3 groups of 3 glands per treatment. Different letters above the columns indicate significant differences among treatments (one way ANOVA p<0.05, with Tukey test of multiple comparisons).

To test if the nutritional status modifies the responsiveness of CA to insulin stimulation *in vitro*, CAs were dissected from 4-day old starved and sugar-fed females and cultured for 4 h in the presence of bovine insulin (17 µM). JH synthesis was significantly stimulated by insulin in glands dissected from starved females; while CA dissected from sugar-fed females did not respond to insulin stimulation (Fig. 6). The addition of insulin increased JH synthesis by CA of starved females to levels equivalent to those produced by glands of sugar-fed females.

## Discussion

### Starvation decreases JH synthesis and reduces nutrient allocation into the ovaries

Reproductive trade-offs in adult insects occur mostly through the process of oosorption. By resorbing excess reproductive tissues, insects can adjust previous allocation decisions by redirecting resources away from reproduction in favor of competing physiological activities [16], [17]. A substantial number of studies have shown that sugar feeding is critical for building energy reserves and improving egg production in a female mosquito [18], [19]. Deprivation of a sugar meal had dramatic effects on nutrient stores; starvation for 3 days resulted in a 10 fold decrease in total lipid reserves. We have previously described that similar starvation conditions resulted in a 4 fold decrease in oocyte lipid content and a 4 fold increase in the rate of resorption [8]; however application of methoprene (a JH analog) to starved females increased stored ovarian lipids and decreased resorption [8].

On the other hand, lipids reserves increased more than 2-fold by feeding 20% sugar for the first 24 h after emergence, indicating that a sugar meal could rapidly augment lipid stores. It is important for a female mosquito to rapidly and effectively detect these dynamic changes in nutrient reserves and correct nutrient allocations. The interactions between the ISP and JH controlling reproductive trade-offs are well documented in insects [5]. The activity of the ISP is directly modulated by the nutritional and feeding state [20]. The *A. aegypti* genome encodes eight insulin-like peptides (ILPs), with three of them (ILP1, ILP3 and ILP8) specifically expressed in brains of adult females [21]. To modulate JH synthesis, the ILPs could either be directly delivered to the CA by projections of the *corpora cardiaca* (CC) or they are received from the circulating hemolymph. Transcript levels for several *A. aegypti* ILPs show age dependent and diet dependent changes in female mosquitoes [22]. While JH is the gonadotrophic hormone responsible for primarily controlling nutrient allocation into the ovary [7], [8],the current findings establish that the starvation-induced reduction in JH biosynthesis is the result of a decrease in insulin signaling and it can be totally reversed by supplying exogenous insulin.

### Starvation decreases insulin signaling and increases insulin sensitivity without affecting CA competence to synthesize JH

The TOR and ILP signaling pathways are considered as nutritional sensors at the cellular and systemic level, respectively. We have previously established that application of bovine insulin on the mosquito CA incubated *in vitro* caused a strong and fast stimulation of JH synthesis [13]. In addition, either systemic depletion of TOR by RNAi or rapamycin administration had inhibitory effects on JH synthesis [13].Furthermore, rapamycin impeded insulin stimulation of JH synthesis in the CA of mosquitoes, confirming that TOR activity is critical for the transduction of insulin signaling [13]. Our new results provide evidence that starvation decreases insulin signaling in the CA and reduces JH synthesis. We detected significant increases in CA transcript levels of some key components of the ISP, such as FOXO, 4E-BP and INSr that are well known markers for the starvation response [23]. FOXO proteins are indispensable in the response to starvation, since they promote conservation of energy and catabolism. FOXO is a major coordinator of the transcriptional response to nutrients downstream of insulin; remarkably, 995 nutrient-responsive genes were regulated by activated FOXO in Drosophila [24]. Among those critical genes are 4E-BP and INSr. We observed a transcriptional activation of the translational repressor 4E-BP in the CA. A transcriptional and post-translational regulation of this TOR effector, finely tuned to the nutritional requirements, was previously described in the fat body of female mosquitoes [25].

**Figure 7:**
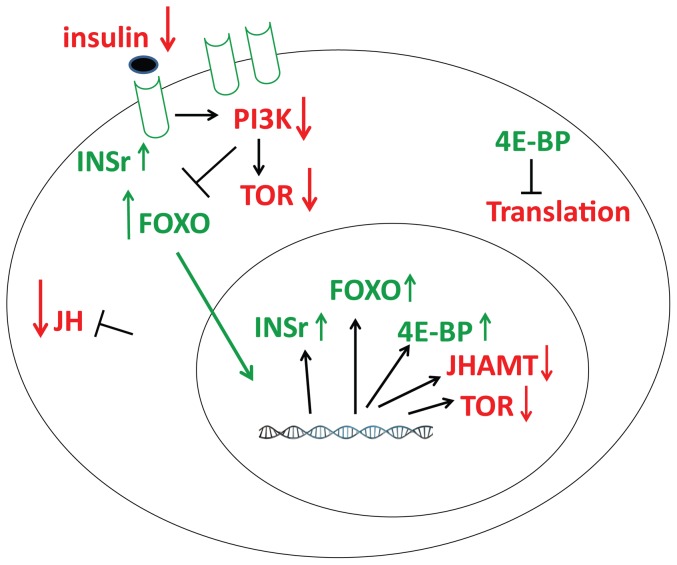
Starvation effects on insulin signaling components and JH synthesis in the CA of mosquitoes. This scheme summarizes starvation-related changes of insulin/TOR pathway components and JH synthesis. Molecules in red color are down-regulated (↓), while those in green are upregulated (↑). Previous experiments established the involvement of phosphoinositide 3-kinase (PI3K) and TOR in the transduction of insulin signaling in the CA [13]. Astarvation-dependent decrease of insulin results in an increase of FOXO signaling that promotes activation of transcription of insulin receptor (INSr) and 4E-binding protein (4EBP). Transcripts levels for FOXO increase and mRNAs for JHAMT and TOR decrease. JH synthesis decrease, while increases of 4EBP inhibit translation and increases of INSr enhance insulin sensitivity.

The insulin receptor itself is a target gene for FOXO regulation in Drosophila and mammals [23], revealing a novel feedback mechanism in the ISP. In this way the insulin receptor can regulate its own synthesis in response to nutrient availability through the modulation of FOXO activity. When nutrients are abundant, FOXO is turned off and downregulation of insulin receptor occurs. However, when nutrients become limited, insulin receptor synthesis becomes upregulated by FOXO and a higher density of receptor molecules accumulate in the cell membrane. The cells thus become “primed” and highly sensitized to respond rapidly to changes in insulin, which signal changes in nutrients [23]. In our studies starvation also significantly increased the expression of the INSr in CA. Our data support a simple model that describes the relationship among starvation, decrease in insulin signaling and decrease in JH synthesis (Fig. 7).

There is evidence that the ISP affects JH levels; in Drosophila, a mutation of the INSr resulted in a significant decrease in JH synthesis, reduced reproductive output and extended life-span [26], [27]. Systemic INSr and TOR RNAi decreased JH contents in honey bee [28]. In termites, insulin signaling was increased by JH application, suggesting that there is a feedback loop connecting JH and insulin signaling and that the ISP may also act downstream of JH [29]. Interplays between insulin signaling, JH and environmental cues (including nutrition) were also described in ants [30]. On the other hand, a direct effect of manipulation of the ISP in the CA has been only described in mosquitoes and cockroaches. Systemic TOR knockdown phenocopies starvation and decreases JH synthesis in the cockroach *Blatella germanica* [31]. In addition, FOXO knockdown in starved cockroaches elicited an increase of JH biosynthesis; implying that FOXO plays an inhibitory role on JH biosynthesis during starvation [32].

In female adult mosquito, the interactions between JH, insulin, nutrition and oogenesis are diverse depending on the tissue and physiological stage. After adult emergence, JH connects the nutritional status with insulin signaling by inducing the expression of TOR signaling components in the fat body. Thus, through JH action, the fat body becomes competent to respond to insulin and transduce the nutritional signal from a blood meal to activate vitellogenin transcription [33]. Later, during vitellogenesis, ILP3 dose-dependently stimulates yolk uptake by oocytes and ecdysteroid production by the mosquito ovaries. ILP3 also exhibits metabolic activity by elevating carbohydrate and lipid storage [10]. *In vivo* knockdown of the mosquito insulin receptor by RNAi delayed but did not fully inhibit trypsin-like gene expression in the midgut, ecdysteroid production by ovaries and vitellogenin expression by the fat body. In contrast, *in vivo* treatment with double-stranded INSr RNA and rapamycin completely blocked egg production [34].

Manipulations that decrease systemically the activity of nutrient sensing system are often difficult to interpret. Depletion of INSr or FOXO has profound consequences beyond reducing the activity of any of these particular genes. We observed that silencing of FOXO very efficiently reduced both FOXO and INSr mRNAs in starved and sugar-fed adult females. That explains that although starvation clearly increased FOXO transcript levels in the CA with a concomitant decrease in JH synthesis, systemic silencing of FOXO decreased INSr levels, phenocopied starvation and reduced JH synthesis in both sugar-fed and starved females. Interestingly, systemic silencing of the INSr had opposite effects in JH synthesis by CA dissected from sugar-fed and starved females. Insulin stimulates JH synthesis, so a decrease in the biosynthetic activity by the CA of sugar-fed females deficient in INSr is expected. On the other hand, the significant increase in JH synthesis by the CA of starved females deficient in INSr might be explained by a systemic depletion of INSr in tissues such as brain, fat body or ovaries that might affect organismal nutrient sensing and feed back into CA physiology.

JHsynthesis by the CA of female mosquitoes displays remarkable dynamic changes. The CA needs to adjust its synthetic activity to generate these dynamic changes [11], [12], [35]. A coordinated expression of most JH biosynthetic enzymes has been described in female pupae and adult mosquitoes; increases or decreases in transcript levels for all the enzymes were concurrent with increases or decreases in JH synthesis [35]. The rate of JH biosynthesis is controlled by the rate of flux of isoprenoids in the pathway, which is the outcome of a complex interplay of changes in precursor pools, enzymatic activity levels and external regulators [12], [35], [36], [37], [38], [39]. The spontaneous rate of JH synthesis is always markedly lower that the maximum inducible rate observed when the CA is stimulated with precursors or activators such as insulin [35], [13]. Reducing insulin signaling with PI3K inhibitors rapidly reduced JH biosynthetic enzyme transcripts; validating that a transcriptional regulation of the genes encoding JH biosynthetic enzymes is at least partially responsible for the dynamic changes of JH biosynthesis [13]. Under starvation conditions, the CA showed a reduction of transcript levels for JHAMT, but the synthetic potential of the CA remained unaffected and the gland rapidly and efficiently responded to insulin stimulation by increasing JH synthesis to rates similar to those of CA from non-starved females.

In summary our studies are providing new insights into the regulatory circuitry that controls both JH synthesis and reproductive output in response to nutrient availability. The decrease of JH synthesis in response to starvation is primarily caused by a reduction on insulin signaling and therefore can be completely reversed by stimulation with insulin. JH primarily controls nutrient allocation into the ovaries and starvation reduces JH synthesis and redirects resources away from reproduction. The CA of starved females remains fully competent to synthesize JH; starvation triggers the up regulation of INSr mRNA in CA cells, so they became more sensitive to insulin signaling, providing a mechanism to amplify insulin signals by allowing their detection in lower concentrations once nutrients are acquired and JH synthesis needs to be reactivated.

## Supporting Information

Table S1 **Primers used for amplification, quantification of the genes studied mRNA and to make dsRNA.**
(DOCX)
