# Correction: Starvation Increases Insulin Sensitivity and Reduces Juvenile Hormone Synthesis in Mosquitoes

**DOI:** 10.1371/journal.pone.0097054

**Published:** 2014-04-30

**Authors:** The PLOS ONE Staff

There are errors throughout Figures 1, 2, 3, and 4. The corrected figures with appropriate asterisks are available here.

**Figure 1:**
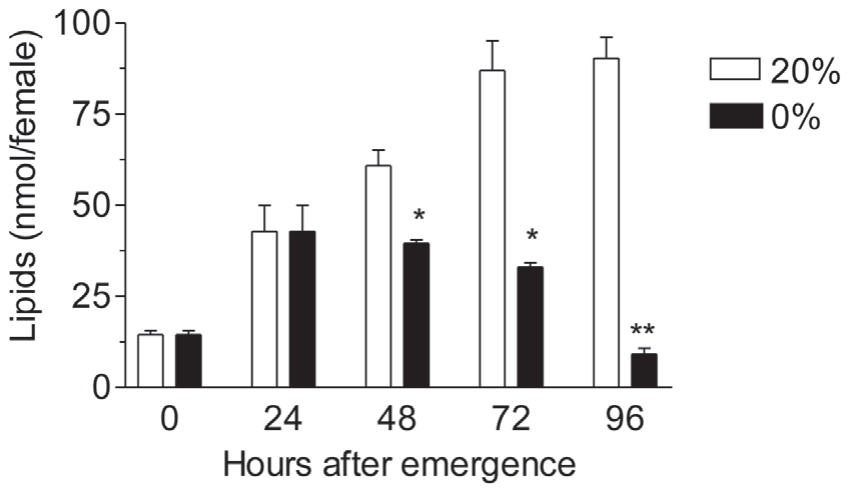
Starvation decreases mosquito lipid reserves. The total amount of lipids in the female body significantly increased when mosquitoes were fed 20% sucrose as compared to those fed water alone (0%). Bars represent the means ±SEM of 3 groups of 5 females per treatment. Asterisks denote significant differences between the two treatments for each time point (unpaired t-test, **P<0.01, *P<0.05).

**Figure 2:**
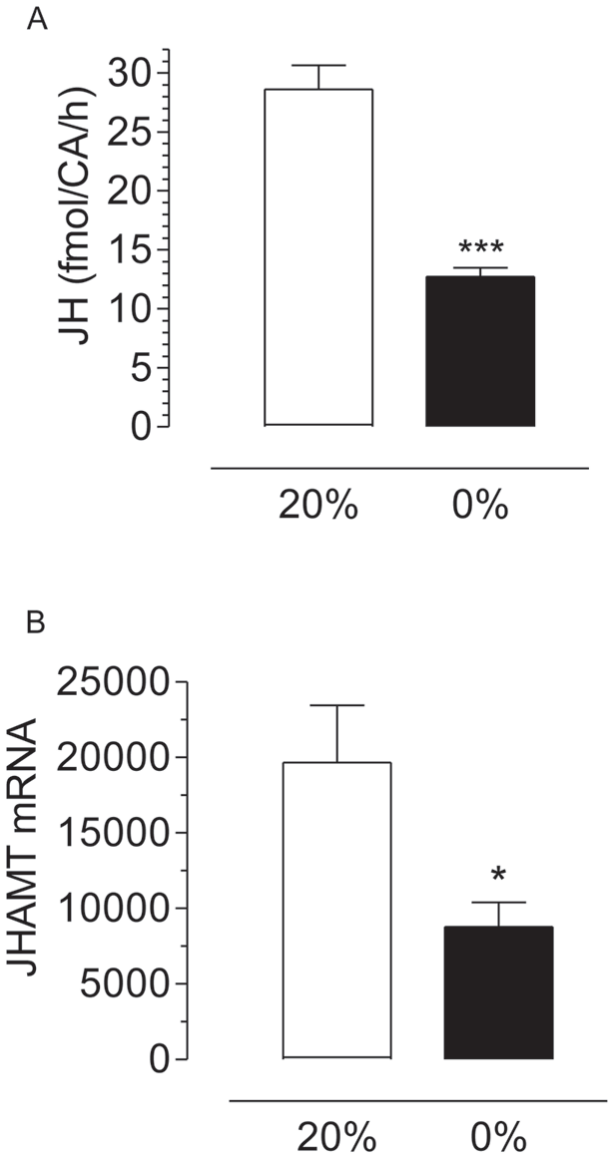
Starvation decreases JH synthesis and JHAMT transcript levels. CA-CC complexes were dissected from 4 days old females after 3 days feeding water (0%) or 20% sucrose (20%). A) JH synthesis: groups of 3 glands were incubated for 4 h; JH synthesis was evaluated by HPLC-FD and it is expressed as fmol per CA per hour. Bars represent the means ± S.E.M. of the analysis of 7 independent experiments of 5 groups of 3 glands per treatment. Different letters above the columns indicate significant differences among treatments (unpaired t-test, ***P<0.001). B) JHAMT transcript levels: CA-CC complexes were dissected from 4 days old females fed on water or 20% sucrose. Transcript levels were measured by qPCR and are expressed as copy number of mRNA JHAMT/10,000 copies of rpL32 mRNA. Bars represent the means ± S.E.M. of 10 replicates of groups of 10 CA each (unpaired t-test, *P<0.05).

**Figure 3:**
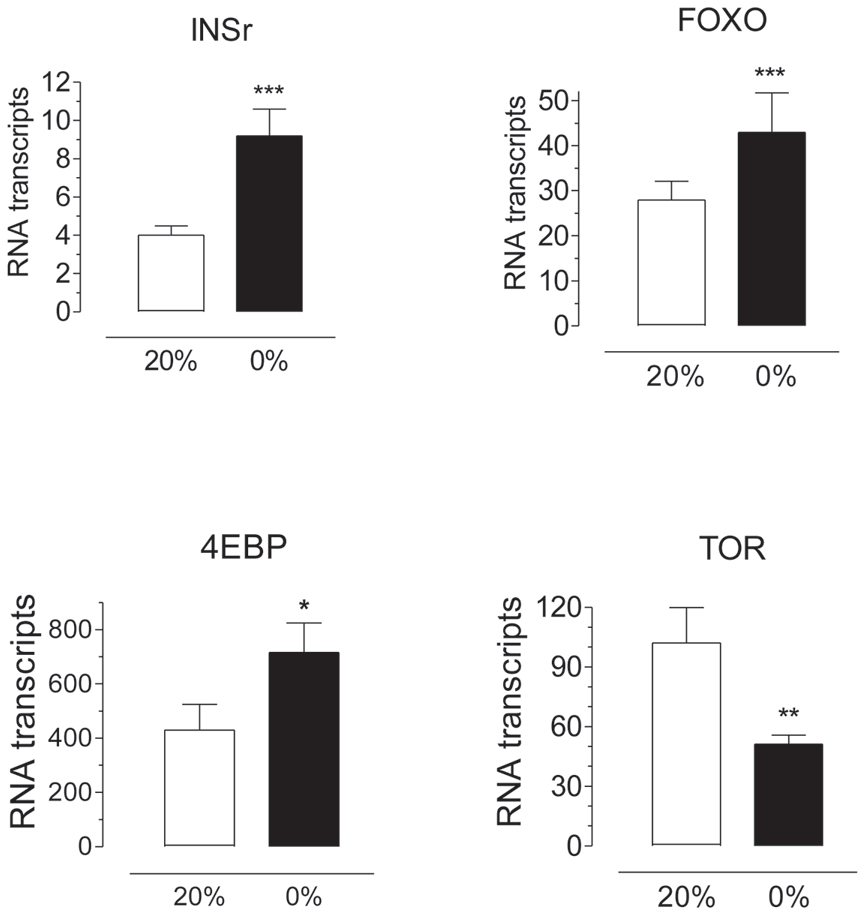
Starvation modulates the expression of insulin/TOR pathway genes. CA-CC complexes were dissected from 4 days old females after 3 days feeding water (0%) or 20% sucrose (20%). Insulin receptor (INSr), forkhead-box 0 (FOXO), 4E-binding protein (4EBP) and target of rapamycin (TOR) transcript levels were measured by qPCR and are expressed as copy number of mRNA gene/10,000 copies of rpL32 mRNA. Bars represent the means ± S.E.M. of 6 replicates of 10 CA each (unpaired t-test, ***P<0.001, **P<0.01, *P<0.05).

**Figure 4:**
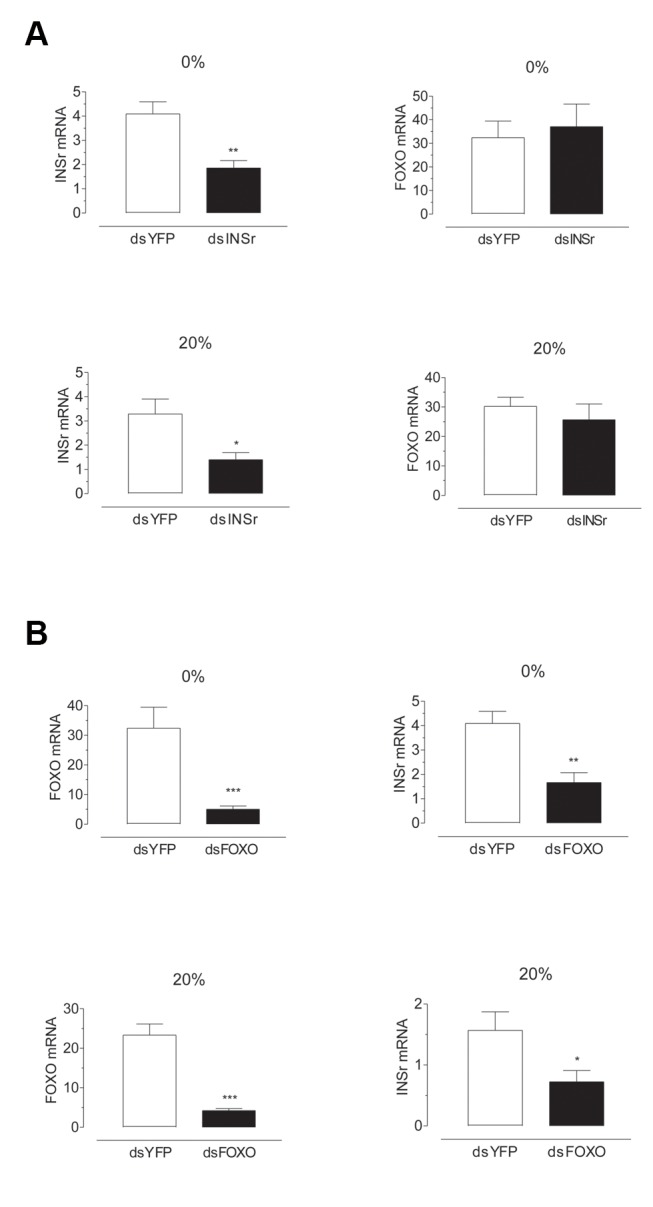
Effects of dsRNA treatment on INSr and FOXO transcript levels. Newly emerged females were injected with dsYFP, dsINSr or dsFOXO. The effect of the RNAi treatment was evaluated 4 days later. A) Effect of dsINSr treatment. B) Effect of dsFOXO treatment. The expression of INSr and FOXO mRNA in the thorax (without legs and wings) were evaluated by qPCR, and it is expressed as copy number of mRNA INSr or FOXO/ 10,000 copies of rpL32 mRNA. Bars represent the means ± S.E.M. of 3 independent biological replicates of 3 groups of 3thoraces. Asterisks denote significant difference (unpaired t-test, ***P<0.001,**P<0.01,*P<0.05).

Please see the corrected Figure 1 here.

Please see the corrected Figure 2 here.

Please see the corrected Figure 3 here.

Please see the corrected Figure 4a and 4b here.
