# Supplementary material for: Starvation Increases Insulin Sensitivity and Reduces Juvenile Hormone Synthesis in Mosquitoes
Source: PLoS One. 2014 Jan 29;9(1):e86183. doi: 10.1371/journal.pone.0086183 (PMC3906049; doi:10.1371/journal.pone.0086183)
Supplement: Table S1 — Primers used for amplification, quantification of the genes studied mRNA and to make dsRNA. (DOCX) [file pone.0086183.s001.docx]

| Gene | Primer forward (5’ → 3’) | Primer reverse (5’ → 3’) | Probe (5’ → 3’) |
| --- | --- | --- | --- |
| rpL32 | CCATCAGTCCGATCGCTATGA | GTTGTCAATACCTTTCGGCTTACG | CAAGCTTGCCCCCAACTG |
| JHAMT | TGCACTGGGTGCGAAGT | CTCCGTTCGGTGCCATAAGATTATA | AAAACGCCGATCTCTG |
| INSr | AAGGATGGCATCTTCAGCAG | GTACCAACGTCACCGATGTG | CCATACCAGGGTCTCACC |
| Foxo | GCGAGTGATACAGCGAACAC | TGAAATCCAGAGATCCTTCCA | TCGATGAAGTGATAAAACA |
| TOR | AAGCTGCTCTGGCTGAAGAG | ATCAGCATTCGCGTCAAAC | TCGCAACCTCGAAGCAA |
| 4E-BP | CTGCCGGATCTCTACTCGTC | TCCATGTCGAACTGCTCTTG | CCAGGAGGTACCCGCATCG |
| INSri | GAAGTGCAATCAGCCATGTG | ATGCCATCCTTCAAGCTTTC | - |
| Fox0i | GCACCAGGACAGGCTCAACT | AACGGTACTGGGCGTAAGTG | - |
| YFPi | AACCGCATCGAGCTGA | ATGGTCAGGCGGGACT | - |
| INSri_T7 | TAATACGACTCACTATAGGGGAAGTGCAATCAGCCATGTG | TAATACGACTCACTATAGGGATGCCATCCTTCAAGCTTTC | - |
| Foxoi_T7 | TAATACGACTCACTATAGGGGCACAAGGACAGGCTCAACT | TAATACGACTCACTATAGGGAACGGTACTGGGCGTAAGTG | - |
| YFPi_T7 | TAATACGACTCACTATAGGGAACCGCATCGAGCTGA | TAATACGACTCACTATAGGGATGGTCAGGCGGGACT | - |

**Table S1.** Primers used for amplification, quantification of the genes studied mRNA and to make dsRNA.
